# Optimized Method for Preparation of IgG-Binding Bacterial Magnetic Nanoparticles

**DOI:** 10.1371/journal.pone.0109914

**Published:** 2014-10-15

**Authors:** Denis S. Grouzdev, Marina V. Dziuba, Denis V. Kurek, Alexander I. Ovchinnikov, Nadezhda A. Zhigalova, Boris B. Kuznetsov, Konstantin G. Skryabin

**Affiliations:** 1 Faculty of Biology, Moscow State University, Moscow, Russia; 2 Centre Bioengineering, Russian Academy of Sciences, Moscow, Russia; George Mason University, United States of America

## Abstract

In this study, the optimized method for designing IgG-binding magnetosomes based on integration of IgG-binding fusion proteins into magnetosome membrane *in vitro* is presented. Fusion proteins Mbb and Mistbb consisting of magnetosome membrane protein MamC and membrane associating protein Mistic from *Bacillus subtilis* as anchors and BB-domains of *Staphylococcus aureus* protein A as IgG-binding region were used. With Response Surface Methodology (RSM) the highest level of proteins integration into magnetosome membrane was achieved under the following parameters: pH 8.78, without adding NaCl and 55 s of vortexing for Mbb; pH 9.48, 323 mM NaCl and 55 s of vortexing for Mistbb. Modified magnetosomes with Mbb and Mistbb displayed on their surface demonstrated comparable levels of IgG-binding activity, suggesting that both proteins could be efficiently used as anchor molecules. We also demonstrated that such modified magnetosomes are stable in PBS buffer during at least two weeks. IgG-binding magnetosomes obtained by this approach could serve as a multifunctional platform for displaying various types of antibodies.

## Introduction

The systems of antibodies conjugated to the surface of magnetic nanoparticles (MNPs) are increasingly used in diagnostics and therapy. Many studies have previously demonstrated their efficiency for cancer cell detection, magnetic separation of stem cells, magnetic immunoassay and as a carrier for targeted drug delivery [1], [2]. Recently, an interesting alternative to these synthetic MNP, called magnetosomes, was found in magnetotactic bacteria. Magnetosomes are intracellular magnetic crystals produced by magnetotactic bacteria (MTB) and also referred to as bacterial magnetic nanoparticles (BMPs) [3], [4]. The advantages of magnetosomes in comparison with artificial MNPs are: i) uniform species-specific size (30–120 nm) and shape; ii) magnetic crystal is coated with a lipoprotein membrane, making BMPs easily dispersed in aqueous suspension and providing an opportunity to modify a surface by genetic engineering; iii) high crystallinity; iv) low cytotoxicity [5], [6]. Due to these features, magnetosomes attract significant interest as biogenic MNPs, which could be used in a number of biomedical applications. For instance, magnetosome chains were shown to be highly efficient for cancer therapy when they are exposed to an alternative magnetic field [7], magnetosomes have been proposed as potential carriers for drugs in tumor treatment and for DNA in genetic transformation [8],[9].

Three general approaches have been proposed to magnetosomal membrane modification: subsequent chemical alterations of purified magnetosomes [10], [11], transformation of MTB with genetic constructs encoding magnetosome membrane proteins fused to foreign proteins (*in vivo* modification) [12]–[14] and insertion of recombinant fusion proteins into magnetosomal membrane *in vitro* [15], [16]. Magnetosome membrane proteins Mms16, MamC, MamF and MamG were proposed as anchor molecules for foreign proteins display on the magnetosome surface during genetic manipulations with MTBs magnetosome. MamC was used in most of such studies as an efficient anchor protein [13], [17], [18]. In GFP-based analysis MamC-GFP displayed the highest expression and fluorescence levels comparing with GFP-tagged magnetosome proteins MamF and MamG. However, genetic manipulations of MTB are still hampered due to the difficulties in cultivation and fastidiousness of these organisms. At the same time, magnetosome membrane proteins can be easily overexpressed in *E. coli* and purified according to the standard procedures, i.e. immobilized metal ion affinity chromatography. Thus, Matsunaga and co-authors have demonstrated insertion of heterologously expressed recombinant MagA-Luc fusion protein consisted of integral magnetosome protein MagA and firefly luciferase into the membrane of purified magnetosomes [16]. This approach seems to be an efficient and simple way for magnetosome surface modification. In this study the role of NaCl concentration and sonication time was investigated but not the mutual influence of such factors as NaCl concentration, pH value and the mode of mechanical action (sonication vs vortexing).

In this study we presented an optimized method for the IgG display on the surface of BMP. Chimeric proteins containing double IgG-binding B-domains of *Staphylococcus aureus* protein A fused with anchor proteins were integrated *in vitro* into the membrane of magnetosomes extracted from the magnetotactic strain *Magnetospirillum* sp. SO-1 by means of simple vortexing procedure. Highly hydrophobic and small (12.4 kDa) protein MamC was chosen as an anchor molecule for introduction of fused proteins into magnetosomal membrane. As another promising protein for this purpose was chosen Mistic, an unusual membrane-associated protein (13 kDa) from *Bacillus subtilis* which was recently found to be capable of autonomous integrating into the membrane [19]. For this study, two genetic constructs, mbb and mistbb, coding the fusion proteins, were synthetized. Both constructs contained double B domain of *Staphilococcus aureus* protein A as immunoglobulin-binding region and differed by their membrane-anchoring domains. In mbb it was MamC protein from *Magnetospirillum magnetotacticum* MS-1, the corresponding domain in mistbb was Mistic proteins from *Bacillus subtilis*. Whereas MamC is the commonly used protein for the functionalization of bacterial magnetosomes [13], [20], Mistic protein in this study was used as anchoring domain for this purpose for the first time. In the previously published studies Mistic was used as a fusion partner for the overexpression of histidine kinase receptors (HKR) in *E. coli* [21], eukaryotic membrane protein (pkjDes4) and a prokaryotic membrane protein (pkjLi) in *Lactococcus lactis* [22], as well as for expression of G-protein coupled receptors (GPCR) in *E. coli* [23]. The insertion procedure was optimized using Response Surface Methodology (RSM). IgG-binding activities of magnetosomes modified by fusion proteins contained MamC and Mistic as anchor domains were compared. The selectivity of obtained BMP was demonstrated using embryonic kidney cells extract contained Kaiso/GFP protein and anti-GFP IgG conjugated with IgG-binding BMP. Finally, stability of modified BMP after storage at +4°C was analyzed.

## Materials and Methods

### Cultivation of Magnetotactic Bacteria

The medium for *Magnetospirillum* sp. SO-1 consisted of (per liter of medium): 1 ml mineral solution [24], 0.7 g KH2PO4, 0.5 g sodium succinate, 0.1 g yeast extract, 0.35 g NaNO3, 10 ml 0.01 M ferric citrate, 0.05 g sodium thioglycolate. pH was adjusted to 6.75 with NaOH. The cells were cultivated at 28°C under microaerobic conditions in a 15-L fermenter for 3–4 days.

### Magnetosomes Extraction and Purification

After achieving growth stationary phase *Magnetospirillum* sp. SO-1 cells were centrifuged 10,000 g for 10 min at +4°C, resuspended in 20 mM HEPES buffer, pH 7.4, contained 4 mM EDTA and 0.1 mM phenylmethylsulfonyl fluoride (PMSF) and disrupted by sonication (Sonopuls, Bandelin). Magnetosomes were isolated from disrupted cell fractions using a neodymium-boron (Nd-B) magnetic stand and washed 15 times with 20 mM HEPES buffer, pH 7.4. Finally magnetosomes were resuspended in the same buffer and stored at +4°C. The absence of cellular debris in the preprations of purified magnetosomes was tested by atomic force microscopy. The portion of purified magnetosomes was dried at 105°C and weighted, thus evaluating the concentration of the remaining portion.

### Bacterial Strains and Growth Media

We used *Magnetospirillum magnetotacticum* strain MS-1 (DSM 3856), a *Staphylococcus aureus* strain (VPKM 1899), and *Escherichia coli* strains XL-1 Blue (Stratagene, United States) and BL21 (DE3) (Novagene, United States). A pET23a(+) vector (Novagene, United States) was used for genetic engineering manipulations. *E. coli* XL-1 Blue cells were grown in an LB medium [25]. Expression of recombinant proteins was performed in a TB medium [26]. A solid medium for the cultivation of single *E. coli* colonies on Petri dishes was prepared by adding 2% agar to the LB medium.

### Construction of the pET23a(+)/mbb and pET23a(+)/mistbb Expression Vectors

Preparation of competent *E. coli* cells, transformation of cells with plasmid DNA, isolation of plasmid DNA, hydrolysis with restriction endonucleases, phosphorylation, electrophoresis in agarose gel, and other standard procedures were performed as described in [25] and as recommended by the manufacturers of the enzymes used. pET23a(+)/mbb performed as described previously [27]. PCR fragment of *mistic* gene was obtained from *Bacillus subtilis* genomic DNA using specially designed sequence-modifying oligonucleotide primers MistF 5′-AGAGGAGATAT**CATATG**GGCTTT-3′ and MistR 5′-CAGAACC**GGATCC**TTCTTTTTCTC-3′. After digestion with *Nde*I and *BamH*1 restrictases *mistic* PCR product was cloned into vector pET23a(+)/mbb replacing *mamC* in genetic construct *mbb* [27]. The presence of the histidine tag at the C-terminus of heterologous expressed proteins allows for the easy identification of proteins and their purification on chelating sorbents. XL-1 Blue *E. coli* cells were transformed by the pET23a(+)/mbb and pET23a(+)/mistbb recombinant plasmids. Clones harboring plasmids with target inserts were selected using PCR screening and subsequent sequencing of inserts using a BigDye Terminator v3.1 Cycle Sequencing Kit (App1ied Biosystems, United States). Nucleotide sequences were determined on an ABI 3730 automatic sequencer (App1ied Biosystems, United States).

### Preparation of Fusion Proteins

BL21 (DE3) *E. coli* cells were transformed by the pET23a(+)/mbb and pET23a(+)/mistbb expression vectors. Expression of genetic constructs was carried out using autoinduction [26].

### Analysis of the Total Protein from BL21 (DE3) *E. coli* Cells

Cells were collected from 1 mL of medium, resuspended in 100 µL of TED buffer (10 mM tris-HCl, pH 6.8, 1 mM EDTA, 1% SDS), and then incubated at 100°C for 5 min. The lysate obtained was analyzed by denaturing electrophoresis in a polyacrylamide gel according to the method of Laemmli. The protein concentration in the solution was determined by the method of Bradford; BSA solutions were used to build a calibration curve.

### Fractionation of Soluble Cellular Proteins of *E. coli*

Cells harvested from 1 mL of medium were resuspended in 50 µL of buffer I (100 mM Tris-HCl, pH 8.0, 0.5 M sucrose, 0.5 mM EDTA). Phenylmethylsulfonyl fluoride (PMSF) was added to the solution to a final concentration of 0.1 mM; 2.5 µL of 2 mg/ml lysozyme solution in the same buffer was also added. The mixture was incubated at room temperature for 20 min, and then 100 µL of buffer I and 100 µL of water were added, mixed, and incubated for 10 min. An equal volume of 0.2% aqueous solution of Triton X-100 was added, and the mixture was incubated for 1 h at room temperature. The cell suspension was frozen at −20°C, thawed at room temperature three times, and then centrifuged at 12,000 g for 3 min. The supernatant contained the soluble protein fraction, and the pellet contained the insoluble protein fraction. The precipitate was resuspended in 100 µL of lysis buffer supplemented with Triton X-100 (to a final concentration of 0.1%). A membrane fraction was obtained in the following way: 150 mL of overnight culture was centrifuged at 12,000 g for 10 min. Cells were suspended in 15 mL of buffer I, and 3 mL of 2 mg/mL lysozyme solution in the same buffer was added to the suspension. The mixture was incubated at room temperature for 30 min, and then 135 mL of buffer I, 150 µL of 0.1 mM PMSF, and 300 µL of 0.5 M EDTA were added to it. The sample was sonicated for 10 min using a Sonopuls UW2070 device (Bandelin, Germany) at a frequency of 20 kHz. The cell debris was removed by centrifugation at 6,000 g for 30 min, and the membrane fraction was precipitated by supernatant centrifugation at 100,000 g for 2 h at +4°C.

### Purification of Fusion Proteins

The presence of histidine tag in both fusion proteins substantially allowed to perform their purification by metal chelate affinity chromatography. The membrane fraction preparation obtained was resuspended in buffer A (20 mM Tris-HCl, pH 8.0, 500 mM NaCl, 5% glycerol, 10 mM β-mercaptoethanol, 10 mM imidazole, 2 mM PMSF, 1.5% lauryl sarcosine) and incubated at room temperature for 1 h. The solubilized membrane fraction was loaded on a Ni-NTA agarose sorbent (Invitrogen, United States) preequilibrated with buffer A. The sorbent was rinsed with three or more volumes of buffer A and then with three volumes of buffer B (20 mM Tris-HCl, pH 8.0, 1 M NaCl, 5% glycerol, 5 mM imidazole, 1% lauryl sarcosin). The target protein was eluted with buffer C (20 mM Tris-HCl, pH 7.5, 130 mM NaCl, 5% glycerol, 500 mM imidazole, 0.5% lauryl sarcosine). The eluate was dialyzed overnight at +4°C against a buffer containing 20 mM Tris-HCl, pH 7.5, 50 mM NaCl, 10% glycerol, and 14.6 mM lauryl sarcosine. The protein concentration in the solution was determined by the method of Bradford.

### Optimization of Fusion Proteins Insertion into Magnetosome Membrane

The optimization of Mbb and Mistbb insertion into magnetosome membrane was carried out using Response Surface Methodology (RSM). Variation due to model inadequacy was evaluated by Lack-of-fit test. The analysis of variance (ANOVA), which was carried out by Fisher's statistical test, was employed for the determination of the significance of the models. The quality of the model was evaluated by the coefficient R^2^. Several parameters possessing the significant influence on the integration of fusion proteins in the magnetosome membrane were tested in a series of pilot experiments: pH value, NaCl concentration, and the type of stirring of suspension (vortexing or sonication). The respective levels of fusion protein integration vs coded levels for the factors are listed in Table 1. Counting data were obtained using Minitab 15.0. The graphical representations of the regression model plots and their corresponding contour plots were obtained using Design-Expert software (version 9.0.1.0, Stat-Ease Inc., USA). In all experiments 10 µg of magnetosomes and 50 µg of each fusion protein were used. Total volume of reaction mixture was 1 ml.

**Table 1:** The level of variables for the Box-Behnken design.

		Level		
Variables	Code	−1	0	1
pH	A	5.0	8.0	11.0
NaCl (mM)	B	0	250	500
Sonication or Vortex (s)	C	5	30	55

### Atomic Force Microscopy

Visualization and size of biogenic magnetic nanoparticles were performed on NTEGRA Prima microscope (NT-MDT, Russia). Semicontact mode was selected for scanning, NSG01 probes (resonant frequency 87–230 kHz, force constant 1,45–15,1 N/m)were used, scanning speed was 1 Hz. 10 µl of sample was incubated on freshly cleaved mica for 1 min which was subsequently rinsed thoroughly with milliQ water and dehydrated in a dry air stream. The resulted preparations were scanned immediately after drying at room temperature and ambient humidity with no additional treatment.

### Expression and Purification of Fusion Protein Kaiso-GFP

The expression vector for methyl–DNA–binding protein Kaiso was generated by inserting human cDNA Zbtb33 (Kaiso) [28] into the pFLAG-CMV-2 contained GFP at the C-termini. Human embryonic kidney (HEK 293) cells were transiently transfected with pFLAG-Kaiso-GFP using *Calcium phosphate* transfection method (Promega) according to the manufacturer's protocol, and cells were used for experiments after 48 h. The nuclear localization of protein Kaiso and transfection efficiency were confirmed using immunofluorescence assay. Cells were seeded on a 24-well, transfected of Kaiso-GFP and after 48 h fixed with 4% paraformaldehyde (15 min, 37°C), and washed with PBS (1×). The preparations were mounted in Mounting medium for fluorescence with DAPI (VECTOR, USA) and visualized on an Olympus BX51 microscope (Olympus, Japan). Then the nuclear extraction was prepared from transfected HEK 293 cells according to standart protocol.

### Enzyme-linked Immunosorbent Assay

The comparison of IgG-binding activities of Mbb and Mistbb was performed by ELISA. Human insulin (1 mg/well) was pre-adsorbed in the wells of the ELISA plate overnight at +4°C. The residual sorption was blocked by incubation with a 1.5% solution of BSA in a PBS-Tween buffer (PBS, 0.05% Tween 20) for 1 h (200 µl/well). Monoclonal mouse IgG antibodies (0.1 µg/well) against human insulin (Imtek, Russia) were added to the wells and incubated for 1 h at room temperature. The wells were washed four times with PBS-Tween buffer, and then the fusion proteins were added at the pre-requisite dilutions and incubated for 1 h at room temperature. After a similar washing procedure, the plates were incubated with 0.1 µg/well of mouse IgG antibodies against the His-tag (Imtek, Russia) for 1 h and detection was performed using a hydrogen peroxide/horseradish peroxidase detection system with TMB (Sigma, USA) as a chromogenic substrate. The adrenocorticotropic hormone carrying a His-tag at the C-terminus was used as a negative control. All assays were carried out in at least triplicate.

### IgG-binding Activity Assay

The ability of modified magnetosomes to bind IgG was tested by magnetic ELISA. 10 µg/well BMP-Mbb or BMP-Mistbb was incubated with 100 µl of horseradish peroxidase-labeled rabbit IgG (100 µg/ml) for 1 h at room temperature. Then magnetosomes were separated on magnetic stand (Promega, USA) and washed 5 times with 100 µl PBST (PBS, 0.05% Tween 20, pH 7.0). Washed magnetosomes were incubated with TMB Liquid Substrate System for ELISA (Sigma-Aldrich, USA) for 1 min, the reaction was terminated by 50 µl 1 M HCl solution. Wild-type magnetosomes were used as negative control. All assays were carried out in at least triplicate.

## Results and Discussion

### Magnetosome Extraction

**Figure 1:**
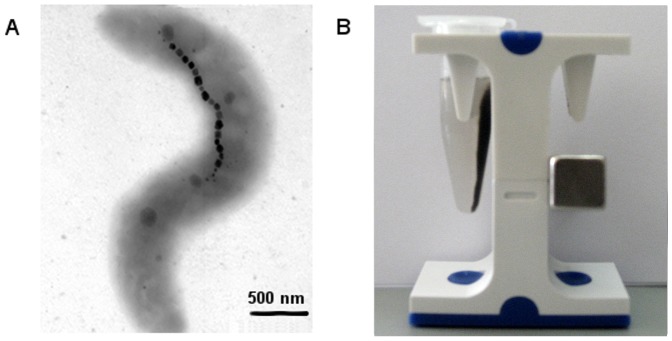
TEM images of *Magnetospirillum* sp. SO-1 (A) and purified magnetosomes on magnetic stand (B). The bar scales are given at the bottom.

Magnetosomes were extracted from cells of Magnetospirillum sp. SO-1, the strain recently isolated from freshwater sediments of the Olkhovka river, Caucasus, Russia (Figure 1) [29]. Magnetosome yield was about 15 mg/l (dry weight).

### Comparison of IgG-binding Activities of Mistbb and Mbb

**Figure 2:**
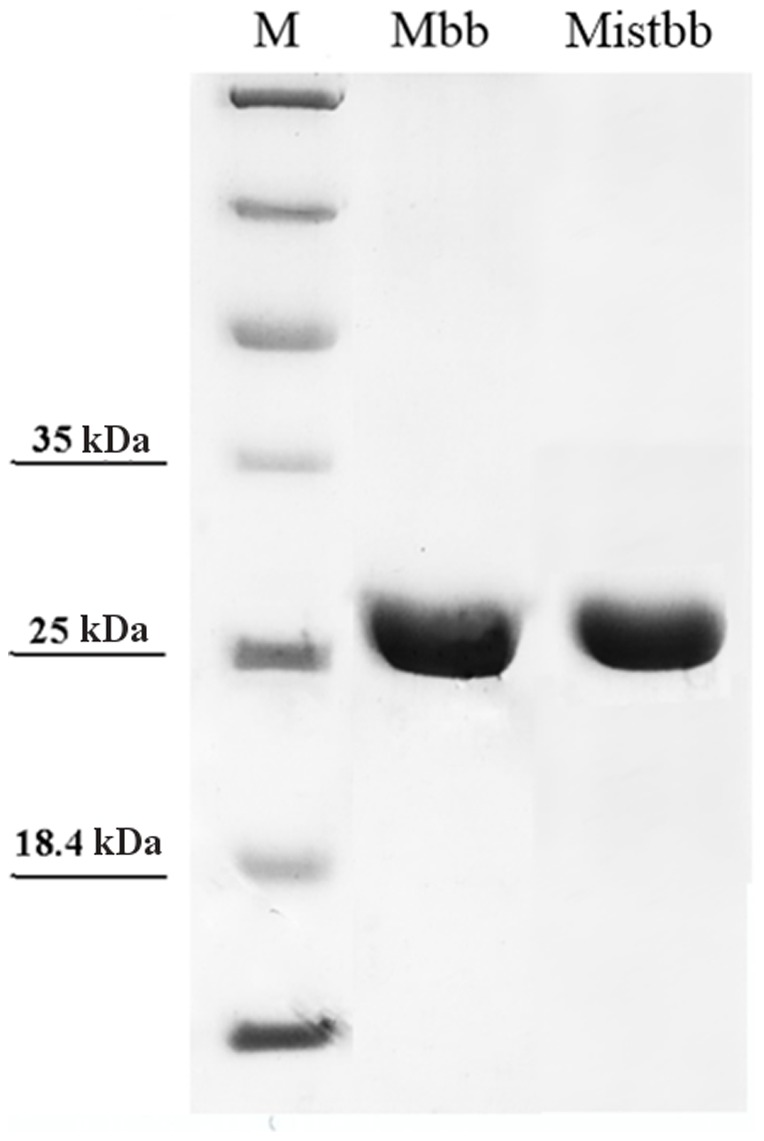
Coomassie stained SDS-PAGE of purified, heterologously expressed proteins Mbb and Mistbb. M – protein molecular weight marker.

**Figure 3:**
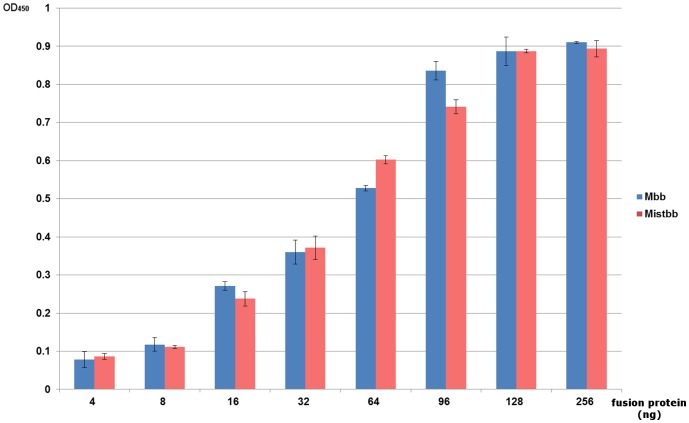
The results of IgG-binding activities of Mistbb and Mbb comparison by ELISA.

After the fusion proteins have been expressed and purified (Figure 2), we compared the IgG-binding activities of Mistbb and Mbb by ELISA. Taking into account, that B-domain of staphylococcal protein A is preferentially bonded with IgG Fc fragment [30], human insulin was absorbed at the surface of immunoplate wells and then primary mouse anti-insulin antibodies were bonded with the absorbed insulin thus providing the proper orientation of their Fc fragments on the well surface. The results of detection of fusion proteins (Mbb or Mistbb) coupled with Fc fragments of primary antibodies are shown at Figure 3. According to the ELISA data, Mistbb exhibited IgG-binding activity similar to Mbb. For the negative control sample no signal was observed. Thereby both proteins were used in further experiments.

### Optimization of the Artificial Insertion

For the proof-of-concept, first, Mbb and Mistbb were inserted into magnetosome membrane by sonication in the presence of 300 mM NaCl according to a previously described procedure [16].

**Figure 4:**
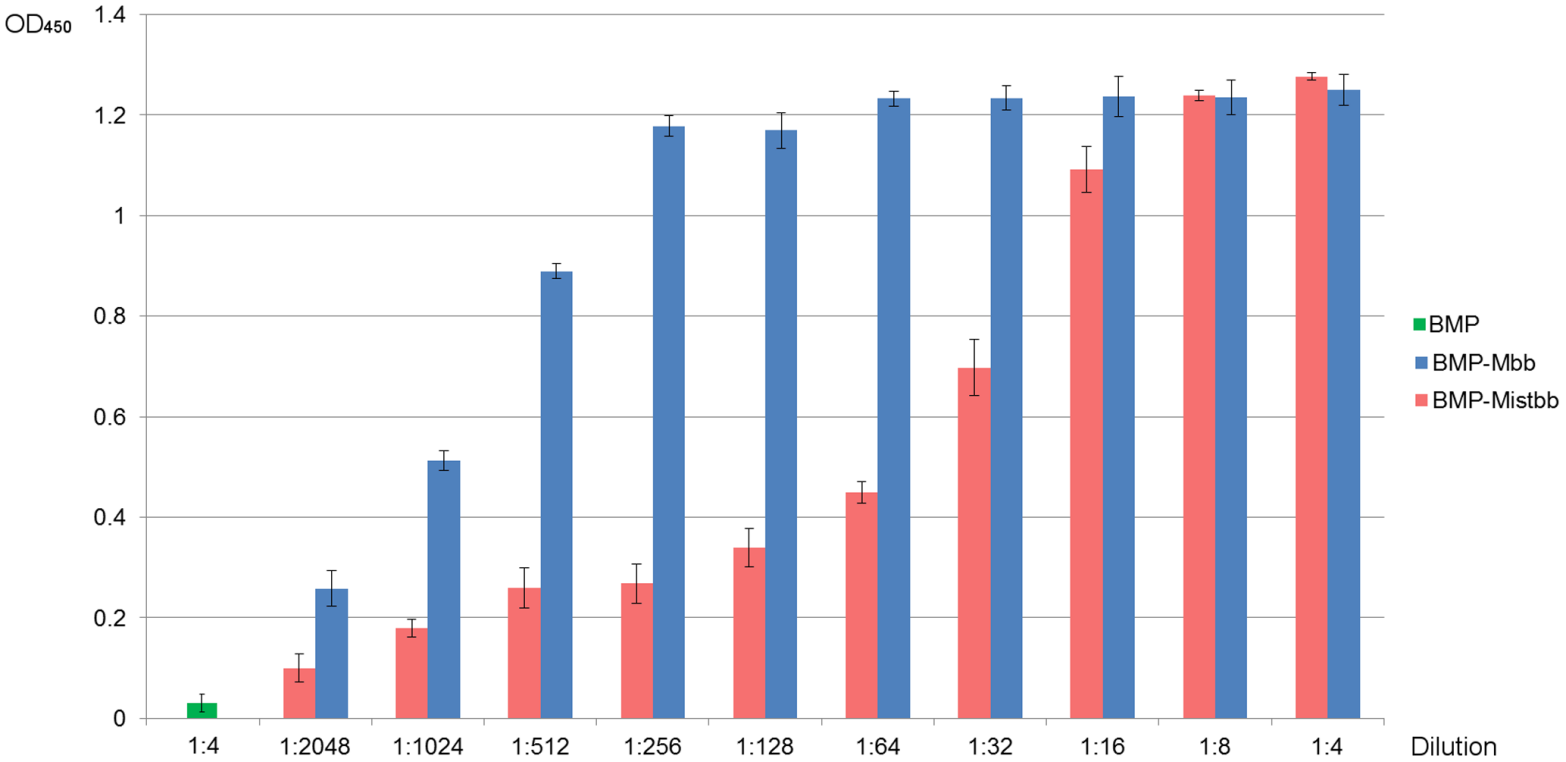
Immunoglobulin-binding ability of Mbb and Mistbb integrated into magnetosome membrane. Dilution 1∶1 corresponds to 1 µg of antibody per 1 ml. Experiment was performed in triplicate.

Immunoglobulin-binding ability of fusion proteins integrated into magnetosome membrane was tested by magnetic ELISA as described in Material and methods section. The data of ELISA are given at Figure 4. As it can be concluded from the data, both proteins kept their ability to bind immunoglobulin, but the IgG-binding activity of magnetosomes with membrane-integrated Mbb protein was higher. When wild-type magnetosomes incubated with antibodies OD_450_ values were significantly low, that indicated negligible non-specific adsorption of antibodies on the surface of BMPs.

As the result of preliminary testing (data not shown), the following parameters affecting the integration of fusion proteins into the magnetosome membrane were chosen - pH, NaCl concentration and mechanical mode of integration (vortexing or sonication). The Response Surface Methodology (RSM) with Box-Behnken design (BBD) was employed to determine the optimal values of selected parameters. It was proved, that this methodology is an effective tool for prediction of optimal process condition for multiple parameter systems [31]–[33]. The respective levels with the coded levels for the factors are listed in Table 1.

The capacity of BMP-Mbb and BMP-Mistbb to bind IgG was tested in assay with horseradish peroxidase-labeled rabbit IgG. OD_450_ values were used for activity rating. Experimental design and results are shown in Table 2. The highest OD_450_ for Mbb was observed under the following conditions – with no NaCl added, pH 8.0, 55 s of vortexing or sonication. In the case of Mistbb integration the highest OD_450_ was achieved for sonication mode with no NaCl added, pH 8.0, 5 s sonication and for vortexing – under 250 mM NaCl, pH 11.0 and 55 s vortexing.

**Table 2:** The matrix of the BBD experiment for optimization of proteins integration and the corresponding experimental data.

Level of variables			Results of magnetic ELISA, OD_450_			
A	B	C	Mbb/sonication	Mistbb/sonication	Mbb/vortexing	Mistbb/vortexing
1	0	1	0.365	0.208	0.598	0.994
1	0	−1	0.203	0.567	0.184	0.129
0	−1	1	1.132	0.168	1.158	0.779
−1	0	1	0.258	0.181	0.465	0.232
0	1	−1	0.982	0.16	0.681	0.297
−1	0	−1	0.841	0.167	0.469	0.134
0	0	0	0.441	0.296	0.752	1.066
1	1	0	0.206	0.57	0.242	0.634
−1	1	0	0.493	0.108	0.931	0.155
0	1	1	0.347	0.41	0.452	0.793
1	−1	0	0.319	0.377	0.961	0.240
0	0	0	0.548	0.157	0.874	0.808
0	−1	−1	0.708	1.01	0.536	0.371
0	0	0	0.473	0.256	0.84	0.817
−1	−1	0	0.258	0.439	0.168	0.265

### RSM Analysis of Mbb Protein Integration through Sonication

The second-order polynomial was as follow:
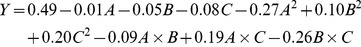
(1.1)


Here Y stands for OD_450_; 0.49 is the intercept; −0.01, −0.05 and −0.08 are the linear coefficients; −0.27, 0.10 and 0.20 are the quadratic coefficients; 0.09, 0.19, −0.26 are the interactive coefficients; and A, B and C are the pH, concentrations of NaCl, and time of sonication.

The equation was sustainable with R^2^ = 0.937, what means that 93.7% of variations could be explained by the chosen model. The “Lack-of-Fit F-value” of 7.43 implies the Lack of Fit is not significant relative to the pure error. There is a 12.09% chance that a “Lack of Fit F-value” this large could occur due to noise.

**Figure 5:**
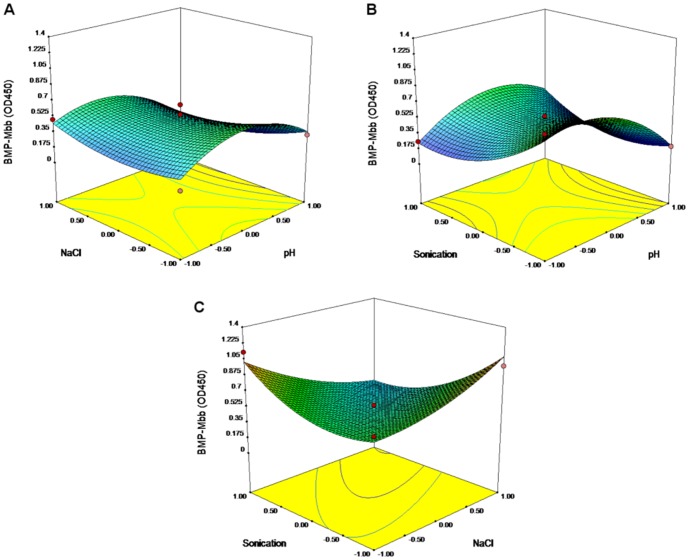
Response surface 3D plots and corresponding contour 2D plots for sonication-mediated integration of Mbb. Combined effects of NaCl concentration and pH level (A); combined effects of pH level and sonication time (B); combined effects of NaCl concentration and time of sonication (C).

To estimate the statistical faithfulness F-test and ANOVA analysis were performed (Table 3). The Model F-value of 8.27 implies the model is significant. There is only a 1.58% chance that an F-value this large could occur due to noise. Between the faithful correlations were: A^2^, C2, A×C, B×C. 3D plots based on equation (1.1) are given at Figure 5.

**Table 3:** The ANOVA results of response surface quadratic model for the Mbb integration by sonication.

Source	DF^b^	SS^c^	*F*-test	*p*-value
Regression	9	1.0904	8.27	0.016^a^
Linear	3	0.1404	3.20	0.122
A	1	0.0716	4.89	0.078
B	1	0.0189	1.29	0.307
C	1	0.0499	3.41	0.124
Square	3	0.5005	11.39	0.011^a^
A×A	1	0.3220	18.63	0.008^a^
B×B	1	0.0288	2.70	0.161
C×C	1	0.1497	10.21	0.024^a^
Interaction	3	0.4494	10.22	0.014^a^
A×B	1	0.0303	2.07	0.210
A×C	1	0.1388	9.47	0.028^a^
B×C	1	0.2804	19.14	0.007^a^
Residual Error	5	0.0733		
Lack of Fit	3	0.0672	7.43	0.121
Pure Error	2	0.0060		
Total	14	1.1637		

a Statistically significant at 95% of confidence level.

b DF, degree of freedom.

c SS, Sum of Squares.

R^2^ = 93.70%.

The optimal predicted parameters for sonication-mediated integration of Mbb protein into magnetosome membrane were the following: pH 5.97, 500 mM NaCl, sonication for 5 s.

### RSM Analysis of Mistbb Protein Integration through Sonication

The second-order polynomial was as follow:
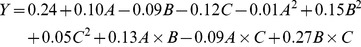
(1.2)


Here Y stands for OD_450_; 0.24 is the intercept; 0.10, −0.09 and −0.12 are the linear coefficients; −0.01, 0.15 and 0.05 are the quadratic coefficients; 0.13, −0.09, 0.27 are the interactive coefficients; and A, B and C are the pH, concentrations of NaCl, and time of sonication.

The equation was sustainable with R^2^ = 0.9431. Lack of fit = 0.317, there is a 31.74% chance that a “Lack of Fit F-value” this large could occur due to noise, however this value is not significant relative to the pure error (Table 4). The Model F-value of 9.20 implies the model is significant. There is only a 1.25% chance that an F-value this large could occur due to noise.

**Table 4:** The ANOVA results of response surface quadratic model for the Mistbb integration by sonication.

Source	DF^b^	SS^c^	F-test	p-value
Regression	9	0.75471	9.20	0.012^a^
Linear	3	0.26480	9.68	0.016^a^
A	1	0.08549	9.38	0.028^a^
B	1	0.06956	7.63	0.04a
C	1	0.10975	12.04	0.018^a^
Square	3	0.08836	3.23	0.12
A×A	1	0.00213	0.04	0.855
B×B	1	0.07548	8.72	0.032^a^
C×C	1	0.01075	1.18	0.327
Interaction	3	0.40154	14.69	0.007^a^
A×B	1	0.06864	7.53	0.041^a^
A×C	1	0.03478	3.82	0.108
B×C	1	0.29812	32.71	0.002^a^
Residual Error	5	0.04557		
Lack of Fit	3	0.03533	2.30	0.317
Pure Error	2	0.01024		
Total	14	0.80028		

a Statistically significant at 95% of confidence level.

b DF, degree of freedom.

c SS, Sum of Squares.

R^2^ = 94.31%.

**Figure 6:**
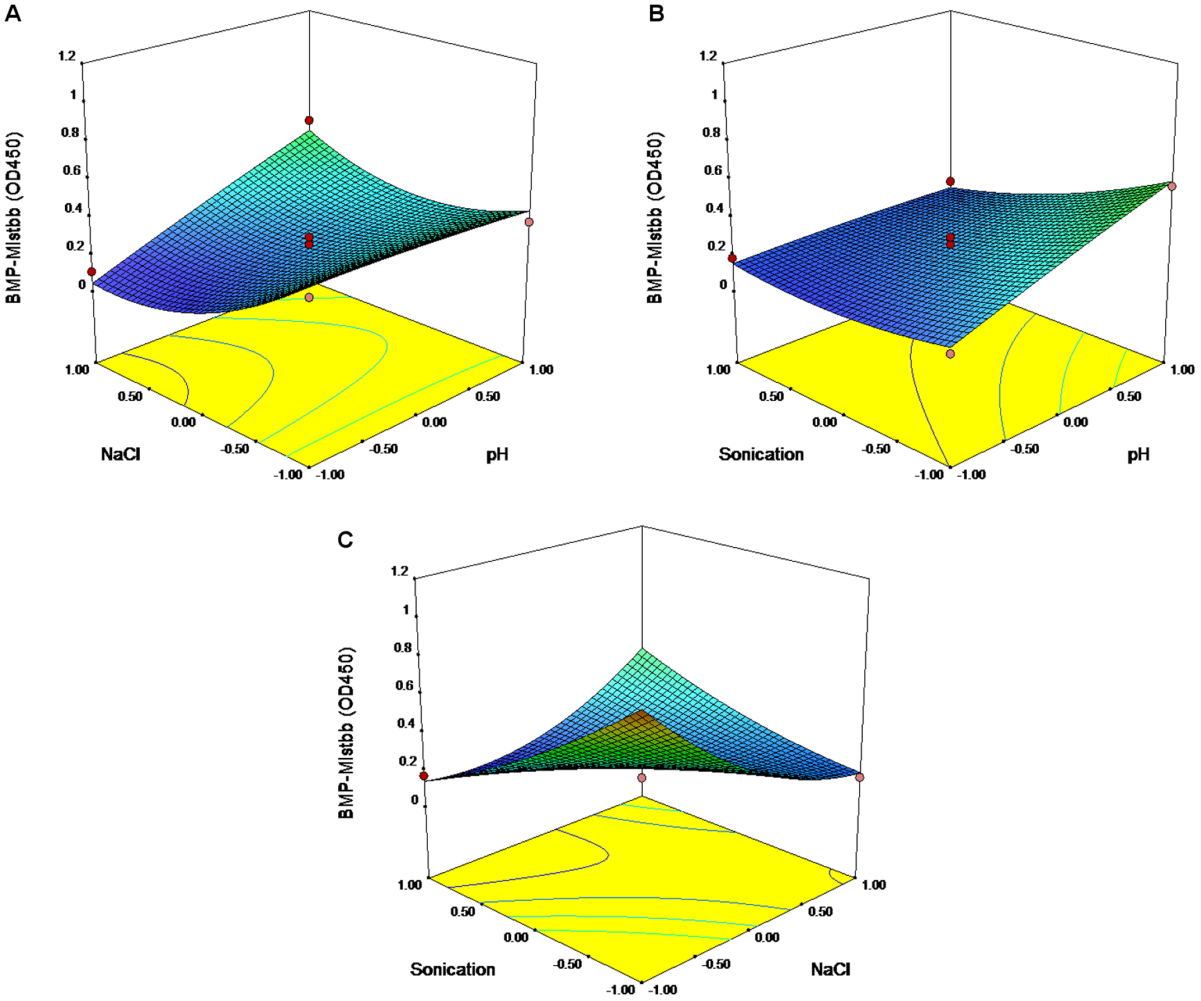
Response surface 3D plots and corresponding contour 2D plots for sonication-mediated integration of Mistbb. Combined effects of NaCl concentration and pH level (A); combined effects of pH level and time of sonication (B); combined effects of NaCl concentration and time of sonication (C).

Between the faithful correlations were: A, B, C, B^2^, A×B, B×C (p-value <0.05). It means that the efficacy of Mistbb integration is effected by (pH value)/(NaCl concentration) and (NaCl concentration)/(sonication time). 3D plots based on equation (1.2) are given in Figure 6. The optimal predicted parameters for ultra-sonic integration of Mistbb protein into magnetosome membrane were the following: pH 8.9, 0 mM NaCl and sonication for 5 s.

### RSM Analysis of Mbb Protein Integration through Vortexing

The second-order polynomial was as follow:
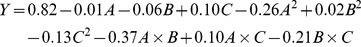
(1.3)


Here Y stands for OD_450_; 0.82 is the intercept; −0.01, −0.06 and 0.10 are the linear coefficients; −0.26, 0.02 and −0.13 are the quadratic coefficients; −0.37, 0.10, −0.21 are the interactive coefficients; and A, B and C are the pH, concentrations of NaCl, and time of vortexing.

**Figure 7:**
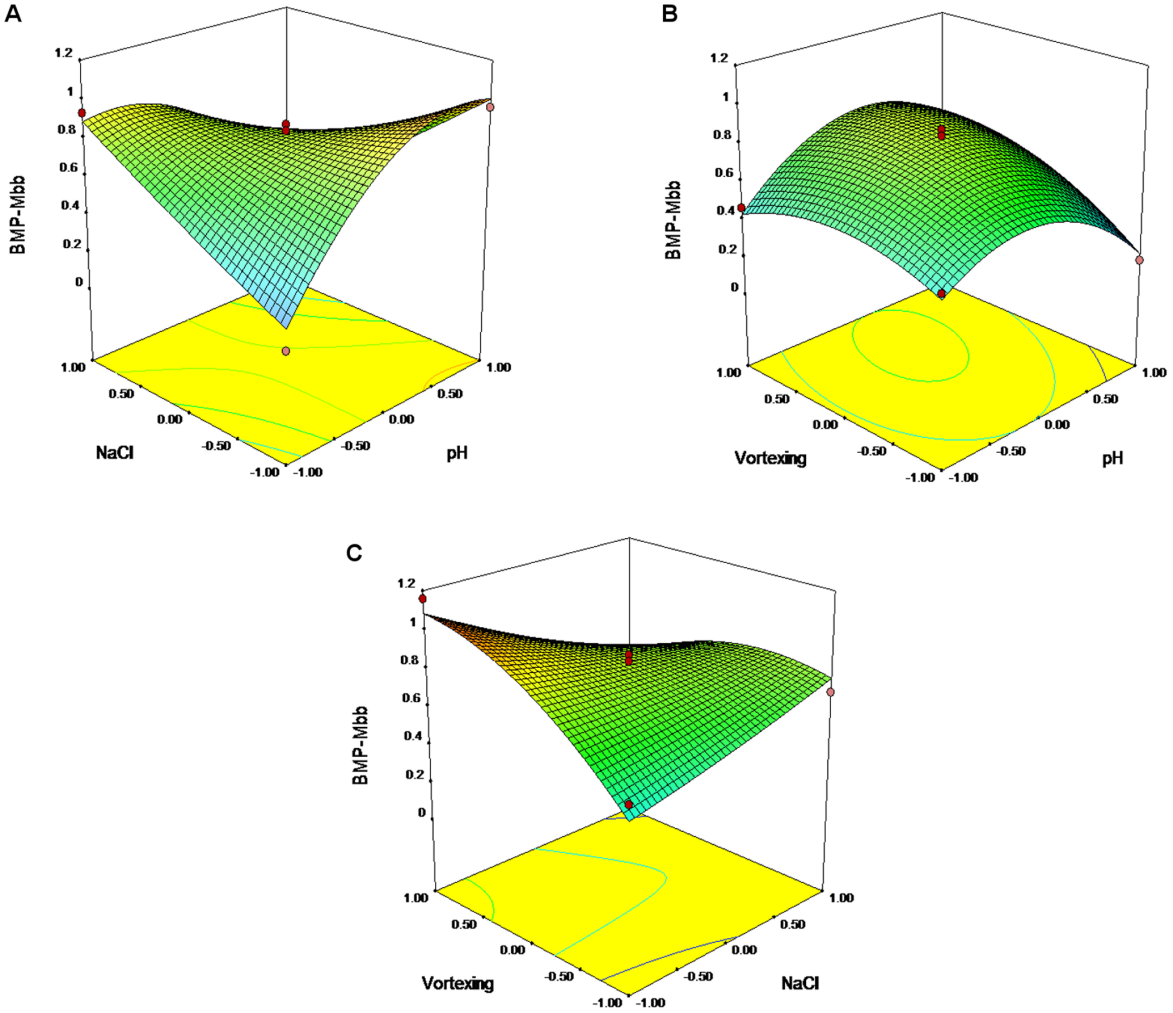
Response surface 3D plots and corresponding contour 2D plots for vortex-mediated integration of Mbb. Combined effects of NaCl concentration and pH level (A); combined effects of pH level and sonication time (B); combined effects of NaCl concentration and time of sonication (C).

The equation was sustainable with R^2^ = 0.9507, lack of fit value = 0.186 supported the adequateness of this model (Table 5). The Model F-value of 10.72 implies the model is significant. There is only a 0.89% chance that an F-value this large could occur due to noise. Between the faithful correlations were: A^2^, A×B, B×C (p-value <0.05). 3D plots based on equation (1.3) are given in Figure 7. The optimal predicted parameters for vortex-mediated integration of Mbb protein into magnetosome membrane were the following: pH 8.78, 0 mM NaCl, 55 s of vortexing.

**Table 5:** The ANOVA results of response surface quadratic model for the Mbb integration by vortexing.

Source	DF^b^	SS^c^	*F*-test	*p*-value
Regression	9	1.1945	10.72	0.009^a^
Linear	3	0.1143	3.08	0.129
A	1	0.0003	0.02	0.885
B	1	0.0334	2.7	0.161
C	1	0.0806	6.51	0.051
Square	3	0.3064	8.25	0.022^a^
A×A	1	0.2407	20.49	0.006^a^
B×B	1	0.0025	0.07	0.798
C×C	1	0.0632	5.11	0.073
Interaction	3	0.7738	20.83	0.003^a^
A×B	1	0.5491	44.35	0.001^a^
A×C	1	0.0437	3.53	0.119
B×C	1	0.1811	14.62	0.012^a^
Residual Error	5	0.0619		
Lack of Fit	3	0.0540	4.54	0.186
Pure Error	2	0.0079		
Total	14	1.2564		

a Statistically significant at 95% of confidence level.

b DF, degree of freedom.

c SS, Sum of Squares.

R^2^ = 95.07%

### RSM Analysis of Mistbb Protein Integration through Vortexing

The second-order polynomial was as follow:
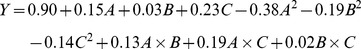
(1.4)


Here Y stands for OD_450_; 0.90 is the intercept; 0.15, 0.03 and 0.23 are the linear coefficients; −0.38, −0.19 and −0.14 are the quadratic coefficients; 0.13, 0.19, 0.02 are the interactive coefficients; and A, B and C are the pH, concentrations of NaCl, and time of vortexing.

**Figure 8:**
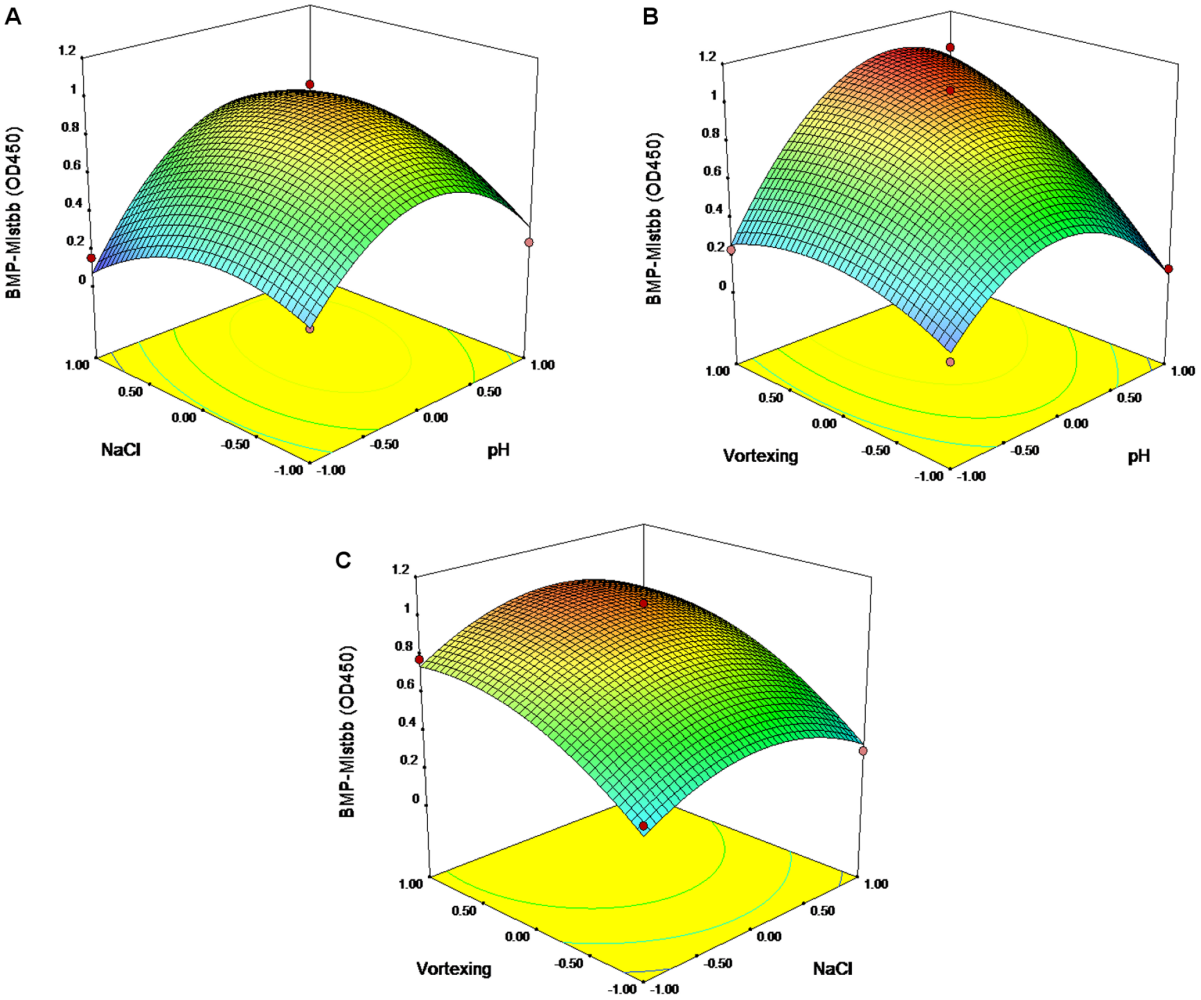
Response surface 3D plots and corresponding contour 2D plots for vortex-mediated integration of Mistbb. Combined effects of NaCl concentration and pH level (A); combined effects of pH level and sonication time (B); combined effects of NaCl concentration and time of sonication (C).

The equation was sustainable with R^2^ = 0.956, lack of fit value = 0.762 supported the adequateness of this model (Table 6). The Model F-value of 10.72 implies the model is significant. There is only a 0.89% chance that an F-value this large could occur due to noise. Between the faithful correlations were: A, C, A^2^, B^2^, A×C. 3D plots based on equation (1.4) are given in Figure 8. The optimal predicted parameters for vortex-mediated integration of Mistbb protein into magnetosome membrane were the following: pH 9.48, 323 mM NaCl, 55 s vortexing.

**Table 6:** The ANOVA results of response surface quadratic model for the Mistbb integration by vortexing.

Source	DF^b^	SS^c^	F	P
Regression	9	1.5119	12.07	0.007^a^
Linear	3	0.6253	14.98	0.006^a^
A	1	0.1833	13.17	0.015^a^
B	1	0.0063	0.45	0.532
C	1	0.4357	31.31	0.003^a^
Square	3	0.6741	16.14	0.005^a^
A×A	1	0.4746	38.44	0.002^a^
B×B	1	0.1228	9.87	0.026^a^
C×C	1	0.0767	5.51	0.066
Interaction	3	0.2125	5.09	0.056
A×B	1	0.0635	4.56	0.086
A×C	1	0.1471	10.57	0.023^a^
B×C	1	0.0019	0.14	0.724
Residual Error	5	0.0696		
Lack of Fit	3	0.0267	0.42	0.762
Pure Error	2	0.0429		
Total	14	1.5815		

a Statistically significant at 95% of confidence level.

b DF, degree of freedom.

c SS, Sum of Squares.

R^2^ = 95.60%.

### Testing the Optimized Conditions

**Figure 9:**
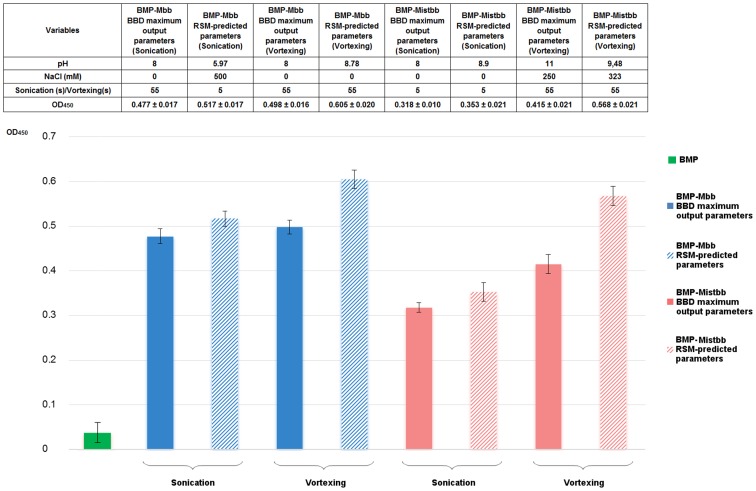
The results of IgG-binding activities of BMP-Mistbb and BMP-Mbb comparison by ELISA. Experiment was performed in triplicate.

To verify the optimal predicted parameters for IgG-binding proteins, a validation experiment was performed in triplicate, where the quantity of magnetosomes per reaction was decreased to 20 µg/well. OD_450_ values obtained with RSM-predicted and BBD maximum output parameters (Figure 9) were compared. According to the results of magnetic ELISA, the reliable difference between both values was detected. The highest OD_450_ values (0.605±0.020 at Mbb and 0.568±0.021 at Mistbb) were achieved after vortex-mediated integration of both proteins under RSM-predicted parameters. Since the absolute levels of integration for Mbb and Mistbb were similar, both proteins are equally applicable as anchor molecules. According to the data obtained under these parameters vortexing was chosen as the most appropriate mode of integration. Since under optimized conditions there were no faithful differences between BMP-Mbb and BMP-Mistbb IgG-binding activities, both proteins could be successfully used for magnetosome membrane modification.

### AFM of Modified Magnetosomes

**Figure 10:**
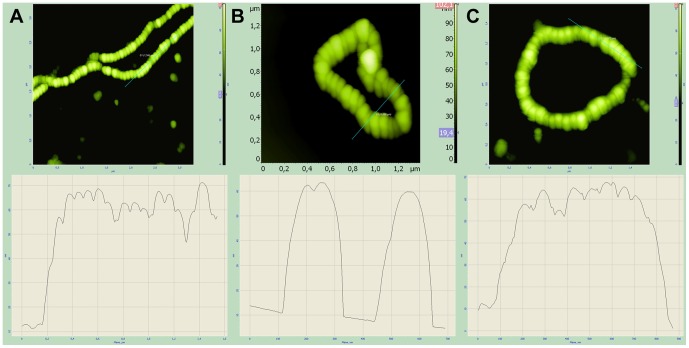
AFM images and topographic cross sections along the lines of intact magnetosomes (A); magnetosomes with integrated Mbb (B) and Mistbb (C) into the magnetosome membrane.

**Figure 11:**
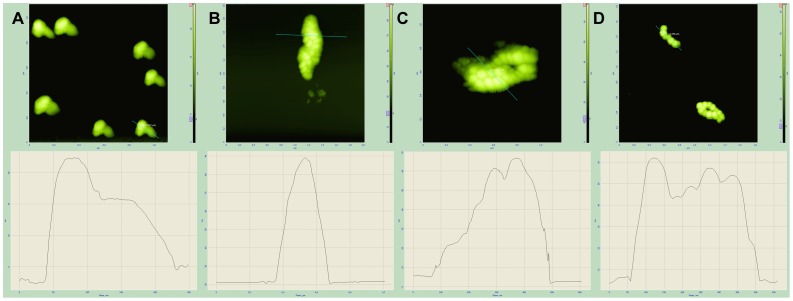
AFM images and topographic cross sections along the lines of IgG (A); BMP-Mbb incubated with IgG (B); BMP-Mistbb incubated with IgG (C); intact magnetosomes incubated with IgG (D).

The morphology of magnetosomes was analyzed by AFM at the various stages of their modification. The intact magnetosomes were 50–60 nm in diameter (Figure 10A). Being conjugated with fusion proteins (Mbb or Mistbb), the resulted magnetosomes possessed the same diameter as intact ones (Figure 10B, C), whereas incubation of fusion protein coupled magnetosomes with IgG their diameter increased up to 95–105 nm and the surface of IgG-bound magnetosome became hilly (Figure 11B, C). Immunoglobulins were seen through AFM as 25 nm knobby corpuscles (Figure 11A).

**Figure 12:**
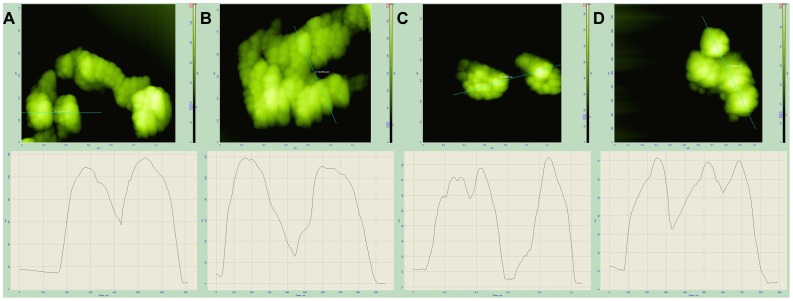
AFM images and topographic cross sections along the lines of BMP-Mbb-anti-GFP IgG (A), BMP-Mistbb-anti-GFP IgG (B). BMP-Mbb-anti-insulin IgG (C), BMP-Mistbb-anti-insulin IgG (D) incubated with HEK 293 nuclear extract.

To examine the ability of modified BMP-IgG to bind selectively to the respective antigen, we used BMP conjugated with anti-GFP IgG and transformed human embryonic kidney (HEK 293) nuclear extract, contained modified Kaiso fused with GFP (Kaiso/GFP). The expression of Kaiso/GFP genetic construction was proven by GFP fluorescence of the transformed cells nuclei (Figure S1). After exposure of IgG-bind magnetosomes conjugated with anti-GFP IgG with Kaiso/GFP containing nuclear extract, magnetosome diameter increased up to 120 nm (Figure 12A, B). No size increase of magnetosomes occurred when wild-type magnetosomes with anti-insulin antibodies immobilized on their surface were incubated with HEK 293 nuclear extract (Figure 12C, D).

### Permanence of BMP-Mbb and BMP-Mistbb

**Figure 13:**
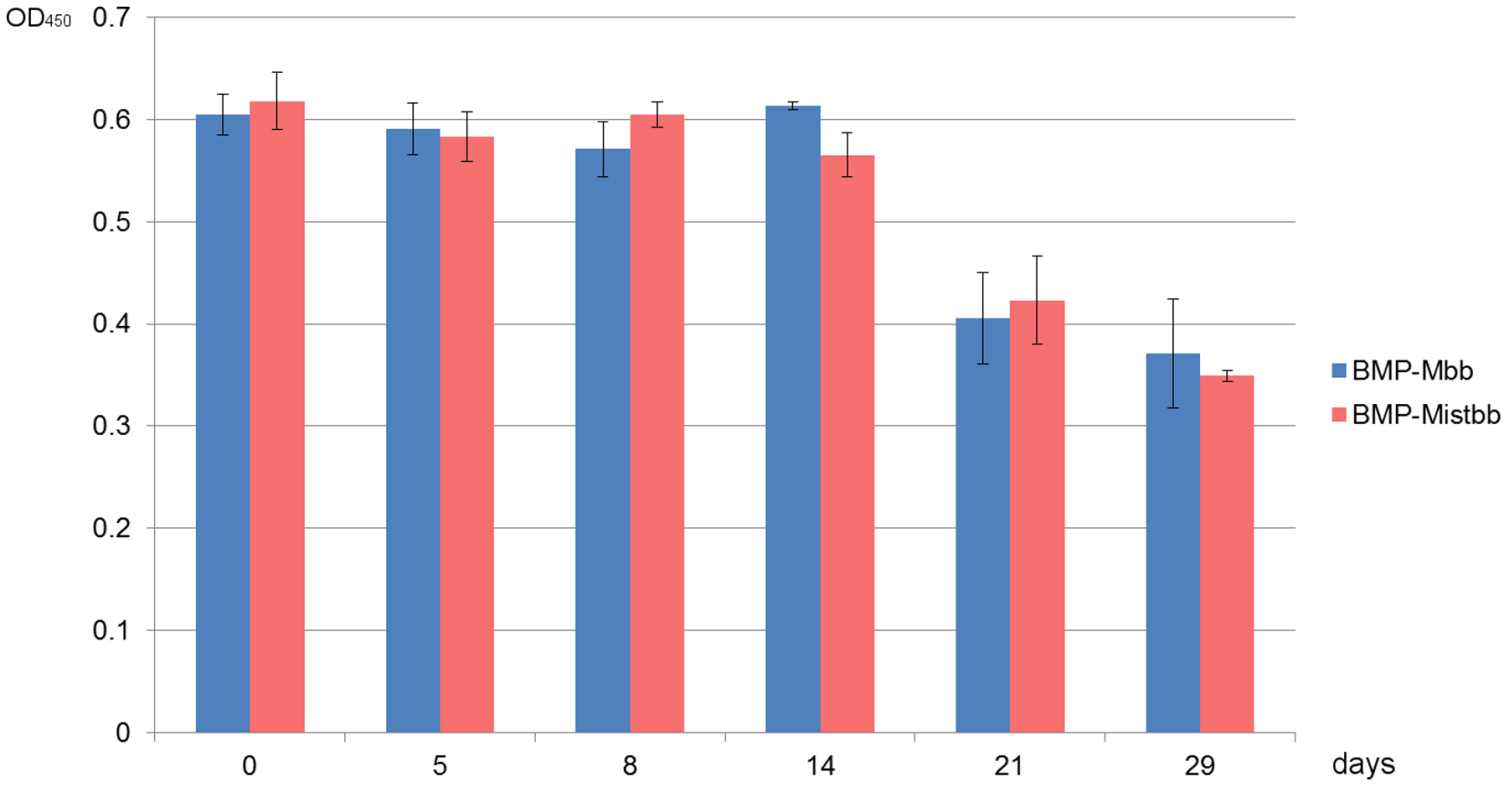
The results of stability testing BMP-Mbb and BMP-Mistbb during long-term storage. Magnetic ELISA data. Experiment was performed in triplicate.

Magnetosomes with Mbb and Mistbb inserted in their membranes were stored in PBS buffer at +4°C for 3 weeks. During this period modified magnetosomes were tested on their ability to bind IgG by magnetic ELISA test. According the results given in Figure 13, BMP-Mbb and BMP-Mistbb retained their IgG-binding activity up to 14 day storage under above conditions. Again, no difference was found between BMP-Mbb and BMP-Mistbb in regard to activity retaining. The decrease of IgG-binding activity at 21 day of storage is presumably accounted for by membrane degradation.

## Conclusion

A simple and efficient procedure for the integration of fusion proteins into magnetosome membrane by vortexing of magnetosome/protein mixture was proposed. The optimal parameters of Mbb integration were determined as follows: pH 8.78, with no NaCl added, and vortexing for 55 s. The highest level of Mistbb integration was achieved at pH 9.48, 323 mM NaCl and 55 s of vortexing. For the first time, we showed that not only original magnetosome membrane proteins but also foreign membrane associating Mistic protein could be effectively used as anchor molecules for integration of hybrid proteins into the BMPs membrane. Also we demonstrated that the resulted modified magnetosomes were stable in PBS buffer for at least two weeks. The optimized procedure for design of IgG-binding BMP *in vitro* could further facilitate the development of methods for functionalizing BMP via protein display on their surface.

## Supporting Information

Figure S1 Fluorescent analysis of human embryonic kidney (HEK 293) cells transiently transfected with pFLAG-Kaiso-GFP. DAPI stained cells (A), GFP fluorescence (B).
(TIFF)

## References

[1] Huh Y-M, Jun Y-w, Song H-T, Kim S, Choi J-s, et al (2005) In vivo magnetic resonance detection of cancer by using multifunctional magnetic nanocrystals. Journal of the American Chemical Society 127: 12387–12391. 16131220 10.1021/ja052337c

[2] Ito A, Shinkai M, Honda H, Kobayashi T (2005) Medical application of functionalized magnetic nanoparticles. Journal of bioscience and bioengineering 100: 1–11. 16233845 10.1263/jbb.100.1

[3] Dunin-Borkowski RE, McCartney MR, Frankel RB, Bazylinski DA, Pósfai M, et al (1998) Magnetic microstructure of magnetotactic bacteria by electron holography. Science 282: 1868–1870. 9836632 10.1126/science.282.5395.1868

[4] Bazylinski DA, Frankel RB (2004) Magnetosome formation in prokaryotes. Nat Rev Microbiol 2: 217–230. 15083157 10.1038/nrmicro842

[5] Moskowitz BM (1995) Biomineralization of magnetic minerals. Reviews of geophysics 33: 123–128.

[6] Xiang L, Wei J, Jianbo S, Guili W, Feng G, et al (2007) Purified and sterilized magnetosomes from Magnetospirillum gryphiswaldense MSR-1 were not toxic to mouse fibroblasts in vitro. Lett Appl Microbiol 45: 75–81. 17594464 10.1111/j.1472-765X.2007.02143.x

[7] Alphandery E, Faure S, Seksek O, Guyot F, Chebbi I (2011) Chains of magnetosomes extracted from AMB-1 magnetotactic bacteria for application in alternative magnetic field cancer therapy. ACS Nano 5: 6279–6296. 21732678 10.1021/nn201290k

[8] Takeyama H, Yamazawa A, Nakamura C, Matsunaga T (1995) Application of bacterial magnetic particles as novel DNA carriers for ballistic transformation of a marine cyanobacterium. Biotechnology techniques 9: 355–360.

[9] Sun JB, Duan JH, Dai SL, Ren J, Zhang YD, et al (2007) In vitro and in vivo antitumor effects of doxorubicin loaded with bacterial magnetosomes (DBMs) on H22 cells: the magnetic bio-nanoparticles as drug carriers. Cancer Lett 258: 109–117. 17920762 10.1016/j.canlet.2007.08.018

[10] Yoza B, Arakaki A, Maruyama K, Takeyama H, Matsunaga T (2003) Fully automated DNA extraction from blood using magnetic particles modified with a hyperbranched polyamidoamine dendrimer. Journal of bioscience and bioengineering 95: 21–26. 16233361 10.1016/S1389-1723(03)80143-3

[11] Ceyhan B, Alhorn P, Lang C, Schuler D, Niemeyer CM (2006) Semisynthetic biogenic magnetosome nanoparticles for the detection of proteins and nucleic acids. Small 2: 1251–1255. 17192969 10.1002/smll.200600282

[12] Matsunaga T, Togo H, Kikuchi T, Tanaka T (2000) Production of luciferase-magnetic particle complex by recombinant Magnetospirillum sp. AMB-1. Biotechnol Bioeng 70: 704–709. 11064341

[13] Lang C, Schuler D (2008) Expression of green fluorescent protein fused to magnetosome proteins in microaerophilic magnetotactic bacteria. Appl Environ Microbiol 74: 4944–4953. 18539817 10.1128/AEM.00231-08 PMC2519370

[14] Wacker R, Ceyhan B, Alhorn P, Schueler D, Lang C, et al (2007) Magneto immuno-PCR: a novel immunoassay based on biogenic magnetosome nanoparticles. Biochem Biophys Res Commun 357: 391–396. 17428442 10.1016/j.bbrc.2007.03.156

[15] Tanaka T, Takeda H, Kokuryu Y, Matsunaga T (2004) Spontaneous integration of transmembrane peptides into a bacterial magnetic particle membrane and its application to display of useful proteins. Analytical chemistry 76: 3764–3769. 15228352 10.1021/ac035361m

[16] Matsunaga T, Arakaki A, Takahoko M (2002) Preparation of luciferase-bacterial magnetic particle complex by artificial integration of MagA-luciferase fusion protein into the bacterial magnetic particle membrane. Biotechnol Bioeng 77: 614–618. 11807756 10.1002/bit.10114

[17] Yoshino T, Matsunaga T (2006) Efficient and stable display of functional proteins on bacterial magnetic particles using mms13 as a novel anchor molecule. Appl Environ Microbiol 72: 465–471. 16391079 10.1128/AEM.72.1.465-471.2006 PMC1352229

[18] Takahashi M, Yoshino T, Matsunaga T (2010) Surface modification of magnetic nanoparticles using asparagines-serine polypeptide designed to control interactions with cell surfaces. Biomaterials 31: 4952–4957. 20363023 10.1016/j.biomaterials.2010.02.048

[19] Roosild TP, Greenwald J, Vega M, Castronovo S, Riek R, et al (2005) NMR structure of Mistic, a membrane-integrating protein for membrane protein expression. Science 307: 1317–1321. 15731457 10.1126/science.1106392

[20] Pollithy A, Romer T, Lang C, Muller FD, Helma J, et al (2011) Magnetosome expression of functional camelid antibody fragments (nanobodies) in Magnetospirillum gryphiswaldense. Appl Environ Microbiol 77: 6165–6171. 21764974 10.1128/AEM.05282-11 PMC3165405

[21] Kefala G, Kwiatkowski W, Esquivies L, Maslennikov I, Choe S (2007) Application of Mistic to improving the expression and membrane integration of histidine kinase receptors from Escherichia coli. Journal of structural and functional genomics 8: 167–172. 17985211 10.1007/s10969-007-9033-4

[22] Xu Y, Kong J, Kong W (2013) Improved membrane protein expression in Lactococcus lactis by fusion to Mistic. Microbiology 159: 1002–1009. 23519161 10.1099/mic.0.066621-0

[23] Petrovskaya L, Shulga A, Bocharova O, Ermolyuk YS, Kryukova E, et al (2010) Expression of G-protein coupled receptors in Escherichia coli for structural studies. Biochemistry (Moscow) 75: 881–891. 20673212 10.1134/s0006297910070102

[24] Pfennig N, Lippert KD (1966) Über das vitamin B12-bedürfnis phototropher Schwefelbakterien. Archiv für Mikrobiologie 55: 245–256.

[25] Sambrook J, Fritsch EF, Maniatis T (1989) Molecular cloning: Cold spring harbor laboratory press New York.

[26] Studier FW (2005) Protein production by auto-induction in high-density shaking cultures. Protein expression and purification 41: 207–234. 15915565 10.1016/j.pep.2005.01.016

[27] Grouzdev D, Dziuba M, Gerasimov A, Kuznetsov B (2013) Production of modified magnetosome membrane proteins and analysis of their activity. Applied Biochemistry and Microbiology 49: 220–226. 10.7868/s0555109913030094 23882942

[28] Prokhortchouk A, Hendrich B, Jørgensen H, Ruzov A, Wilm M, et al (2001) The p120 catenin partner Kaiso is a DNA methylation-dependent transcriptional repressor. Genes & development 15: 1613–1618. 11445535 10.1101/gad.198501 PMC312733

[29] Dziuba M, Kolganova T, Gorlenko V, Kuznetsov B (2013) Species diversity of magnetotactic bacteria from the Ol'khovka River, Russia. Microbiology 82: 335–340. 24466736 10.7868/s0026365613030038

[30] Foster T, Hook M (1998) Surface protein adhesins of Staphylococcus aureus. Trends in Microbiology 6: 484–488. 10036727 10.1016/s0966-842x(98)01400-0

[31] Qiang L, Yumei L, Sheng H, Yingzi L, Dongxue S, et al (2013) Optimization of fermentation conditions and properties of an exopolysaccharide from Klebsiella sp. H-207 and application in adsorption of hexavalent chromium. PloS one 8: e53542. 23320092 10.1371/journal.pone.0053542 PMC3539975

[32] Baş D, Boyacı İH (2007) Modeling and optimization I: Usability of response surface methodology. Journal of Food Engineering 78: 836–845.

[33] Bezerra MA, Santelli RE, Oliveira EP, Villar LS, Escaleira LA (2008) Response surface methodology (RSM) as a tool for optimization in analytical chemistry. Talanta 76: 965–977. 18761143 10.1016/j.talanta.2008.05.019

