# Supplementary figures and images for: Optimized Method for Preparation of IgG-Binding Bacterial Magnetic Nanoparticles
Source: PLoS One. 2014 Oct 15;9(10):e109914. doi: 10.1371/journal.pone.0109914 (PMC4198182; doi:10.1371/journal.pone.0109914)

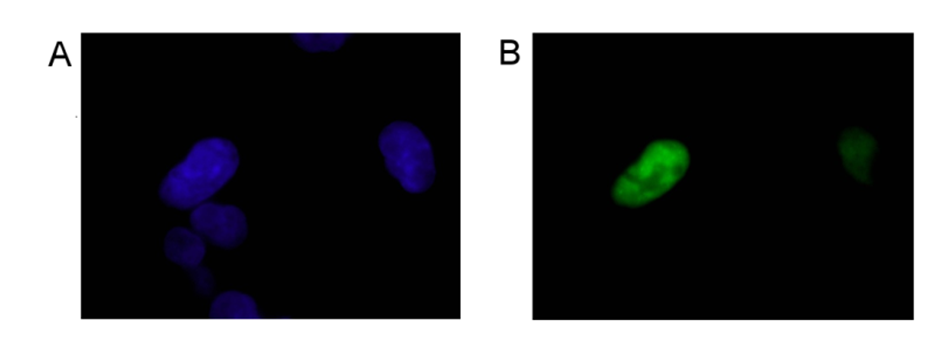

Supplement: Figure S1 — Fluorescent analysis of human embryonic kidney (HEK 293) cells transiently transfected with pFLAG-Kaiso-GFP. DAPI stained cells (A), GFP fluorescence (B). (TIFF) [file pone.0109914.s001.tiff]
